# Loneliness and isolation: Are they associated with the wish for an earlier end of life?

**DOI:** 10.1111/ggi.70148

**Published:** 2025-08-14

**Authors:** André Hajek, Larissa Zwar, Razak M Gyasi, Dong Keon Yon, Supa Pengpid, Karl Peltzer, Hans‐Helmut König

**Affiliations:** ^1^ Department of Health Economics and Health Services Research University Medical Center Hamburg‐Eppendorf, Hamburg Center for Health Economics Hamburg Germany; ^2^ Department of Psychology Brock University St. Catharines Ontario Canada; ^3^ African Population and Health Research Center Nairobi Kenya; ^4^ National Center for Naturopathic Medicine, Faculty of Health Southern Cross University Lismore New South Wales Australia; ^5^ Center for Digital Health, Medical Science Research Institute Kyung Hee University College of Medicine Seoul South Korea; ^6^ Department of Health Education and Behavioral Sciences, Faculty of Public Health Mahidol University Bangkok Thailand; ^7^ Department of Public Health Sefako Makgatho Health Sciences University Pretoria South Africa; ^8^ Department of Healthcare Administration, College of Medical and Health Science Asia University Taichung Taiwan; ^9^ Department of Psychology University of the Free State Bloemfontein South Africa; ^10^ Department of Psychology, College of Medical and Health Science Asia University Taichung Taiwan

**Keywords:** desired age at death, life expectancy, loneliness, social isolation, suicidal ideation, depression, wish to die earlier

## Abstract

**Aim:**

To examine the association of loneliness and social isolation with the desired age at death among middle‐aged and older adults in Germany.

**Methods:**

Data were taken from the German Aging Survey (wave 8: nationally representative sample of community‐dwelling individuals aged ≥43 years; *n* = 3826). The mean age equaled 69.3 years (SD 11.3 years, 43–99 years). Loneliness and social isolation were both quantified using psychometrically sound and widely used tools. Several sociodemographic, lifestyle‐related and health‐related covariates were included in linear regression analysis (with robust standard errors).

**Results:**

The mean desired age at death was 90.1 years (SD 8.0 years). Regressions showed that there was a robust association of loneliness (β = −1.47, *P* < 0.001) and social isolation (β = −1.49, *P* < 0.001) with lower desired age at death among the total sample, even after adjusting for a wide array of covariates. In the fully‐adjusted model, such associations were also present among both men and women (with significant sex differences for the association between loneliness and the outcome; i.e., more pronounced association between loneliness and the desired age at death among men).

**Conclusions:**

Loneliness (among men in particular) and social isolation were both associated with a lower desired age at death. This stresses the importance of tackling loneliness and social isolation in later life. It is of note that this is the very first study examining the association of loneliness and social isolation with the desired age at death. Thus, it can serve as a basis for future studies. **Geriatr Gerontol Int 2025; 25: 1412–1417**.

## Graphical abstract

Loneliness (among men in particular) and social isolation were both associated with a lower desired age at death. Avoiding loneliness and isolation could lead to older people wanting to live longer.

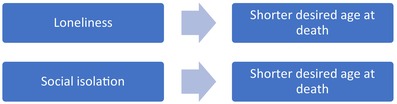

## Introduction

There is an ongoing demographic aging in many countries globally. Various events can take place in old age, such as losing friends and relatives, or loss of functional abilities. Such critical events in life can lead to feelings of loneliness—a perceived discrepancy between one's own desired and actual social relationships—or social isolation—feeling that one does not belong to society.^1^, ^2^, ^3^, ^4^ Such unmet social needs can contribute to various deleterious health outcomes, including morbidity and mortality.^5^, ^6^

Two studies also showed that both loneliness and social isolation are associated with a decrease in expected longevity, and an increased frequency of dealing with death and dying among community‐dwelling individuals in their second half of life in Germany.^7^, ^8^ However, the existing evidence only refers to the expectation of how long one will presumably live. So far, there is no single study on the association of loneliness and isolation with the desired age at death. In contrast to expected longevity, desired age at death also explicitly refers to how old one actually wants to become.^9^ We believe that this is valuable information, because it explicitly refers to the preferences of individuals. Knowing such preferences is also important to better understand and meet the needs of different groups in society—which is also important for health policy, healthcare, financing and resource planning. It can also support the development of public health programs aimed at successful aging. Furthermore, knowledge about the factors associated with desired age at death is of great importance, as such preferences might also have an impact on the current and upcoming lifestyle habits. Ultimately, such habits might also have an effect on future health and successful aging. Furthermore, shorter desired longevity is associated with an increased mortality risk.^10^ Due to the large gap of knowledge and the described relevance of this association, our aim was to investigate the association of loneliness and social isolation with the desired age at death (also stratified by sex).

If individuals tend to perceive their current situation negatively and do not expect good prospects for change (i.e. a more negative future perspective), one could assume that they have a low desired age at death. In this context, we argue that lonely and isolated people, in particular, might report a low desired age at death due to the factors mentioned above. For example, it has been shown that loneliness and isolation are associated with pessimism.^11^, ^12^ Furthermore, individuals scoring high in loneliness and isolation might have a low purpose in life,^13^ ultimately resulting in a low desired age at death. Additionally, lonely and isolated individuals might assume that they would receive little (private) support if they needed care in the future, precisely because of these unmet social needs. Furthermore, because many individuals want to be cared for at home for as long as possible (and thus try to avoid living in a nursing home),^14^, ^15^ this in turn could lead to a shorter desired remaining lifespan. Additionally, lonely and isolated people might also be aware that they will presumably have more chronic illnesses in the future. This could also lead to them not wanting to live as long—to avoid further years of poor health.

We assume that the aforementioned associations are particularly pronounced among men, particularly due to differences in coping between sexes.^16^, ^17^ More specifically, when faced with loneliness and social isolation, women might have better coping strategies (such as discussing such issues with family and friends) to deal with such challenges compared with men.

## Methods

### Sample

Data were taken from wave 8 of the German Aging Survey (DEAS). Data collection was carried out from December 2022 to June 2023. We solely used data from this most recent wave for reasons of data availability; that is, information on the outcome measure was exclusively collected in this wave. A slightly higher number of interviews (~ 56%) were carried out by telephone (computer‐assisted telephone interviews) than in person (computer‐assisted personal interviews; ~44%). In wave 8, the response rate was approximately 62%. Our analytic sample equaled 3826 individuals. A small selection bias was present; that is, the participation rate was significantly positively associated with younger age, higher education, being female and favorable health. However, the rate was mainly not associated with income, marital status, household composition and municipal size category in wave 8.

On average, the interviews lasted approximately 81 min (computer‐assisted telephone interviews 77 min on average; computer‐assisted personal interviews 86 min on average). After finishing the interview, participants could fill out an additional questionnaire with more sensitive topics (e.g. loneliness, social isolation or desired age at death) by post or online. All participants received a thank you letter and a small gift (€15).

Informed consent was provided by all individuals participating in the DEAS study. The DEAS study is in line with the Declaration of Helsinki and its later amendments. An ethics vote was not required, as the criteria for such a vote were not met (e.g. examination of patients or risk to participants).

### Outcome: Desired age at death

Individuals were asked how long they would want to live (when they can decide for themselves). They should specify an exact age in years. A few implausible values were removed (e.g. when the desired age at death was lower than the chronological age).

### Independent variables of interest

Loneliness and social isolation served as key independent variables. The established De Jong Gierveld tool was used to measure loneliness.^18^ Examples of items are as follows: “I experience a general sense of emptiness” or “There are many people I can trust completely.” Three of the six items were recoded before the items were averaged to produce a final score. This score varies from 1 to 4, with higher scores reflecting higher loneliness levels. In this study, Cronbach's alpha was 0.84 (McDonald's omega 0.84).

Furthermore, the Bude and Lantermann tool^19^ (consisting of 4 items) was used to assess social isolation. Exemplary items are: “I feel like I do not really belong to society” or “I feel that I am left out.” By averaging all items, a final score was calculated. This score ranges from 1 to 4, whereby higher values correspond to higher levels of social isolation. In this study, Cronbach's alpha was 0.87 (McDonald's omega 0.87).

### Covariates

A wide array of covariates were included in regression analysis following former research.^7^, ^8^, ^20^, ^21^, ^22^, ^23^, ^24^ Regarding sociodemographic factors, we included age, sex (men, women), marital status (married, living together with spouse; married, living separated from spouse; single; widowed; divorced) and employment status (employed, retired, other), education (International Standard Classification of Education^25^; 3 categories: low [i.e. without a vocational qualification or A‐levels (Abitur)], medium [i.e. either with vocational qualification, or A‐levels without vocational qualification or degrees] or higher education [i.e. with university degrees or master craftsman qualification]).

Regarding lifestyle‐related factors, it was adjusted for the frequency of sports activity (daily; several times a week; once a week; 1–3 times a month; less often; never), smoking behavior (yes, daily; yes, sometimes; no, not anymore, no, never) and alcohol intake (daily; several times a week; once a week; 1–3 times a month; less often; never).

Regarding health‐related factors, it was adjusted for physical functioning, probable depression and chronic conditions. Physical functioning was measured using the SF‐36 subscale physical functioning (based on 10 items).^26^ A total score was built ranging from 0 to 100, whereby higher values reflected better physical functioning. Probable depression was measured by the Center for Epidemiologic Studies Depression Scale (15‐item version^27^; sum score ranges from 0 to 45, with higher values reflecting more depressive symptoms; probable depression was assumed if sum score ≥ 18). A count score of chronic conditions was also used based on the presence/absence of 11 physical diseases (cardiac and circulatory disorders; bad circulation; joint, bone, spinal or back problems; respiratory problems, asthma, shortness of breath; stomach and intestinal problems; cancer; diabetes; gall bladder, liver or kidney problems; bladder problems; eye problems, vision impairment; ear problems, hearing problems).

## Statistical analysis

First, sample characteristics are shown. Then, unadjusted and adjusted linear regressions are calculated. First, it was adjusted for a set of sociodemographic covariates. Then, lifestyle‐related factors were added to the regression model. Finally, health‐related covariates were added to the model. Robust standard errors were calculated. Due to the low proportion of missing values (most of the variables had markedly <1% missing; in total, 3.8% of the observations had at least one missing value for any of the specified variables), listwise deletion was used in the main analysis. A full information maximum likelihood approach was used to check the robustness of our findings.^28^

We used a tool developed by Shaw to compute McDonald's omega.^29^ Statistical significance was defined by a *P*‐value of <0.05. The statistical analyses were carried out using StataNow MP 18.5 (StataCorp, College Station, TX, USA).

## Results

### Sample characteristics

The analytic sample (worth repeating: *n* = 3826 individuals) is shown in Table 1. While the mean age was 69.3 years (SD 11.3 years; 43–99 years), the mean desired age at death was 90.1 years (SD 8.0 years). Overall, 51.6% of the participants were women. The mean loneliness score was 1.7 (SD 0.5) and the mean social isolation score was 1.6 (SD 0.6). More details are shown in Table 1.

**Table 1:** Sample characteristics (analytic sample with *n* = 3826 individuals)

Variables	Mean (SD)/*n* (%)
Current age (years)	69.3 (11.3)
Desired age at death (years)	90.1 (8.0)
Sex	
Men	1853 (48.4%)
Women	1973 (51.6%)
Marital status	
Married, living together with spouse	2567 (67.1%)
Married, living separated from spouse	38 (1.0%)
Divorced	367 (9.6%)
Widowed	575 (15.0%)
Single	279 (7.3%)
Employment status	
Employed	1080 (28.2%)
Retired	2569 (67.1%)
Other: not employed	177 (4.6%)
Education	
Low	127 (3.3%)
Medium	1754 (45.8%)
High	1945 (50.8%)
Smoking behavior	
Yes, daily	390 (10.2%)
Yes, sometimes	150 (3.9%)
No, not anymore	1507 (39.4%)
No, never	1779 (46.5%)
Frequency of sports activity	
Daily	386 (10.1%)
Several times a week	1196 (31.3%)
Once a week	666 (17.4%)
1–3 times a month	221 (5.8%)
Less often	376 (9.8%)
Never	981 (25.6%)
Alcohol intake	
Daily	429 (11.2%)
Several times a week	978 (25.6%)
Once a week	634 (16.6%)
1–3 times a month	514 (13.4%)
Less often	867 (22.7%)
Never	404 (10.6%)
Number of chronic conditions (0–11)	2.7 (2.0)
Physical functioning (0–100, with higher scores reflecting better physical functioning)	80.9 (23.1)
Probable depression	
Absence of probable depression	3593 (93.9%)
Presence of probable depression	233 (6.1%)
Loneliness (1–4, with higher values reflecting higher loneliness levels)	1.7 (0.5)
Social isolation (1–4, with higher values reflecting higher social isolation levels)	1.6 (0.6)

### Regression analysis

Findings of linear regressions (unadjusted and adjusted) for the association of loneliness and social isolation with the desired age at death among the total sample are shown in Table 2 (among men in Table 3; among women in Table 4).

**Table 2:** Association of loneliness and social isolation with the desired age at death among the total sample. Results of linear regressions (wave 8)

	Desired age at death	Desired age at death	Desired age at death	Desired age at death	Desired age at death	Desired age at death	Desired age at death	Desired age at death
Loneliness	−1.69***	−1.79***	−1.77***	−1.47***				
	(−2.20 to −1.18)	(−2.31 to −1.27)	(−2.29 to −1.26)	(−2.01 to −0.93)				
Social isolation					−1.74***	−1.79***	−1.81***	−1.49***
					(−2.20 to −1.29)	(−2.24 to −1.34)	(−2.26 to −1.36)	(−1.97 to −1.01)
Sociodemographic covariates		✓	✓	✓		✓	✓	✓
Lifestyle‐related covariates			✓	✓			✓	✓
Health‐related covariates				✓				✓
Individuals	3940	3927	3896	3826	3940	3927	3893	3824
*R* ^2^	0.01	0.04	0.05	0.05	0.02	0.05	0.05	0.06

Comments: Unstandardized beta coefficients are presented. 95% confidence intervals in parentheses; ***P < 0.001, **P < 0.01, *P < 0.05, +*P* < 0.10.

Sociodemographic covariates include: age, sex, marital status, employment status and education; lifestyle‐related covariates include: smoking behavior, frequency of sports activity and alcohol intake; health‐related covariates include: number of chronic conditions, physical functioning and probable depression.

**Table 3:** Association of loneliness and social isolation with the desired age at death among men. Results of linear regressions (wave 8)

	Desired age at death	Desired age at death	Desired age at death	Desired age at death	Desired age at death	Desired age at death	Desired age at death	Desired age at death
Loneliness	−2.59***	−2.48***	−2.50***	−2.18***				
	(−3.38 to −1.80)	(−3.30 to −1.67)	(−3.31 to −1.69)	(−3.02 to −1.34)				
Social isolation					−2.13***	−2.09***	−2.13***	−1.74***
					(−2.83 to −1.43)	(−2.79 to −1.40)	(−2.82 to −1.43)	(−2.49 to −1.00)
Sociodemographic covariates		✓	✓	✓		✓	✓	✓
Lifestyle‐related covariates			✓	✓			✓	✓
Health‐related covariates				✓				✓
Individuals	1899	1890	1877	1853	1898	1889	1874	1850
*R* ^2^	0.02	0.03	0.05	0.05	0.02	0.03	0.04	0.05

Comments: Unstandardized beta coefficients are presented. 95% confidence intervals in parentheses; ***P < 0.001, **P < 0.01, *P < 0.05, +*P* < 0.10.

Sociodemographic covariates include: age, marital status, employment status and education; lifestyle‐related covariates include: smoking behavior, frequency of sports activity and alcohol intake; health‐related covariates include: number of chronic conditions, physical functioning and probable depression.

**Table 4:** Association of loneliness and social isolation with the desired age at death among women. Results of linear regressions (wave 8)

	Desired age at death	Desired age at death	Desired age at death	Desired age at death	Desired age at death	Desired age at death	Desired age at death	Desired age at death
Loneliness	−1.21***	−1.24***	−1.25***	−1.03**				
	(−1.88 to −0.54)	(−1.92 to −0.56)	(−1.93 to −0.57)	(−1.75 to −0.32)				
Social isolation					−1.36***	−1.43***	−1.49***	−1.33***
					(−1.94 to −0.77)	(−2.01 to −0.84)	(−2.08 to −0.90)	(−1.95 to −0.71)
Sociodemographic covariates		✓	✓	✓		✓	✓	✓
Lifestyle‐related covariates			✓	✓			✓	✓
Health‐related covariates				✓				✓
Individuals	2041	2037	2019	1973	2042	2038	2019	1974
*R* ^2^	0.01	0.01	0.02	0.03	0.01	0.02	0.03	0.03

Comments: Unstandardized beta coefficients are presented. 95% confidence intervals in parentheses; ***P < 0.001, **P < 0.01, *P < 0.05, +*P* < 0.10.

Sociodemographic covariates include: age, marital status, employment status and education; lifestyle‐related covariates include: smoking behavior, frequency of sports activity and alcohol intake; health‐related covariates include: number of chronic conditions, physical functioning and probable depression.

In the fully‐adjusted model (i.e. adjusting for sociodemographic, lifestyle‐related and health‐related covariates), both higher loneliness (β = −1.47, *P* < 0.001) and social isolation (β = −1.49, *P* < 0.001) were still significantly associated with the desired age at death in the total sample. Such associations of loneliness (β = −2.18, *P* < 0.001) and social isolation (β = −1.74, *P* < 0.001) with the desired age at death were particularly pronounced among men. However, the associations of loneliness (β = −1.03, *P* < 0.01) and social isolation (β = −1.33, *P* < 0.001) were also present among women. There was a significant interaction between sex and loneliness (interaction term β = 1.34, *P* = 0.01), whereas the interaction term between sex and perceived social isolation did not reach statistical significance (β = 0.58, *P* = 0.21).

Generally, it is worth noting that particularly adjusting for health‐related covariates attenuated the effect sizes of the associations of loneliness and social isolation with desired age at death among the total sample and both sexes. See Tables 2, 3, 4 for further details. When using a full information maximum likelihood approach (rather than listwise deletion) to handle missing values, findings remained nearly the same in terms of effect size and significance.

## Discussion

The aim of this study was to examine the association of loneliness and social isolation with the desired age at death based on a large, nationally representative sample. Our key findings were as follows: There was a robust association of both loneliness and social isolation with the desired age at death among the total sample, even after adjusting for a wide array of covariates. In the fully‐adjusted model, such associations were also present among men and women (with a significant interaction: sex × loneliness). It is of note that this is the very first study examining the association of loneliness and social isolation with the desired age at death. It can be seen as a foundation for future studies, and hopefully inspire and guide them.

Several factors might explain why both loneliness and social isolation are associated with the wish to die earlier. For example, individuals scoring high in loneliness and social isolation might feel more hopeless, because they might fear that the current situation will not change substantially in upcoming years.^11^, ^12^ They might also lack a meaning in life due to feelings of being excluded from society or missing social interactions, and might thus be less motivated to live.^13^ Furthermore, such feelings might be associated with emotional pain. The perceived lack of social contact might also lead to a lack of reflection, ultimately contributing to a loss of empathy and support. They might feel misunderstood and less accepted, eventually resulting in a shorter desired age at death. They might also fear that if they need care in the future, there will be no family or friends to look after them, which can also contribute to a desire to die earlier.^7^, ^8^

Some factors might explain why the association of loneliness with the desired age at death was more pronounced among men. For example, men are often socialized to show their emotions less openly and to not talk about feelings, such as loneliness.^30^ This might lead to more profound inner torment. Women might have stronger social support and relationships than men.^31^ This might help to cope better with feelings of loneliness. The general self‐esteem of men could also be severely affected by loneliness. Men might miss social recognition, which could be very important to them.^32^ Furthermore, they could compare themselves with other individuals who, from their point of view, are more socially integrated.^33^ This could lead to a downward spiral and a desire for an earlier end to life. However, it should be noted that these are potential explanations that should be further explored in future studies.

When interpreting our findings, several strengths and shortcomings are worth noting. Data were taken from a large sample representative of individuals in their second half of life residing in private households in Germany. Established and valid tools were used to quantify loneliness and social isolation. A wide array of covariates was included in our regression model. A question with high face validity served as the basis for measuring the outcome. The reasons for the desired age at which one would like to live are, however, unknown. The cross‐sectional nature of the data is a shortcoming (particularly in terms of directionality of the findings).

Loneliness (particularly among men) and social isolation were both associated with a shorter desired further lifespan. Such findings stress the relevance of addressing loneliness and social isolation in later life. Future research is required to elucidate the underlying mechanisms. Moreover, studies from other countries (such as Japan) are needed. Furthermore, longitudinal studies are recommended. Other moderating factors (such as general self‐efficacy) could be explored in upcoming studies.

## Funding information

This research received no external funding.

## Disclosure statement

The authors declare no conflict of interest.

## Author contributions

AH: conceptualization, data curation, methodology, project administration, visualization; roles/writing – original draft, writing – review & editing, formal analysis. LZ: conceptualization, writing – review & editing, visualization. RMG: conceptualization, writing – review & editing, visualization. DKY: conceptualization, writing – review & editing, visualization. SP: conceptualization, writing – review & editing, visualization. KP: conceptualization, writing – review & editing, visualization. HHK: conceptualization, resources, writing – review & editing, visualization, supervision. All authors have read and agreed to the published version of the manuscript.

## Ethics statement

All individuals participating in the DEAS study provided informed consent. The study adhered to the Declaration of Helsinki and its subsequent amendments. An ethical review was not required, as the study did not meet the criteria for such a review (for example, it did not involve examination of patients or pose risks to participants).

## Data Availability

The data used in this study are third‐party data. The anonymized datasets of the DEAS (1996, 2002, 2008, 2011, 2014, 2017, 2020, 2020/2021, 2023) are available for secondary analysis. The data have been made available to scientists at universities and research institutes exclusively for scientific purposes. The use of data is subject to written data protection agreements. Microdata of the German Aging Survey (DEAS) are available free of charge to scientific researchers for non‐profitable purposes. The FDZ‐DZA provides access and support to scholars interested in using DEAS for their research. However, for reasons of data protection, signing a data distribution contract is required before data can be obtained. For further information on the data distribution contract, please see https://www.dza.de/en/research/fdz/access-to-data/application (accessed on 8 April 2025).

## References

[1] Pengpid S , Peltzer K , Hajek A , Gyasi RM . Factors associated with loneliness in a national study among persons 80 years and older in India in 2017‐2018. Int Psychogeriatr 2025; 37: 100019. 40086905 10.1016/j.inpsyc.2024.100019

[2] Pengpid S , Peltzer K . Loneliness and associated factors among middle‐aged and older adults: cross‐sectional and longitudinal survey results from the HAALSI cohort in South Africa. Aging Ment Health 2024; 28: 1179–1187. 38726552 10.1080/13607863.2024.2345777

[3] Hajek A , Soysal P , Gyasi RM *et al*. Behind closed doors: Homeboundness and psychosocial outcomes. Evidence from a longitudinal study of middle‐aged and older adults. Arch Gerontol Geriatr 2025; 131: 105767. 39862581 10.1016/j.archger.2025.105767

[4] Petersen N , König H‐H , Hajek A . The link between falls, social isolation and loneliness: a systematic review. Arch Gerontol Geriatr 2020; 88: 104020. 32018091 10.1016/j.archger.2020.104020

[5] Deason KG , Luchetti M , Karakose S *et al*. Neuroticism, loneliness, all‐cause and cause‐specific mortality: a 17‐year study of nearly 500,000 individuals. J Affect Disord 2025; 368: 274–281. 39288835 10.1016/j.jad.2024.09.077 PMC11840298

[6] Jensen MM , Friis K , Maindal HT *et al*. Loneliness is associated with adverse health behaviour and obesity: a Danish population‐based study of 122,258 individuals. BMC Public Health 2025; 25: 375. 39881306 10.1186/s12889-025-21490-4 PMC11781069

[7] Hajek A , König H‐H . Do loneliness and perceived social isolation reduce expected longevity and increase the frequency of dealing with death and dying? Longitudinal findings based on a nationally representative sample. J Am Med Dir Assoc 2021; 22: 1720–1725.e5. 33971163 10.1016/j.jamda.2021.04.004

[8] Hajek A , König HH . Do lonely and socially isolated individuals think they die earlier? The link between loneliness, social isolation and expectations of longevity based on a nationally representative sample. Psychogeriatrics 2021; 21: 571–576. 33966322 10.1111/psyg.12707

[9] Lang FR , Rupprecht FS . Motivation for longevity across the life span: an emerging issue. Innov Aging 2019; 3: igz014. 31240268 10.1093/geroni/igz014 PMC6585880

[10] Yokokawa Y , Sone T , Matsuyama S *et al*. How long would you like to live? A 25‐year prospective observation of the association between desired longevity and mortality. J Epidemiol 2023; 33: 464–470. 35527000 10.2188/jea.JE20210493 PMC10409531

[11] Kupcewicz E , Rachubińska K , Gaworska‐Krzemińska A *et al*. Loneliness and optimism among polish nursing students during the covid‐19 pandemic: the mediatory role of self‐efficacy. Healthcare 2022 10: 971. 35742023 10.3390/healthcare10060971 PMC9222995

[12] Craig H , Gasevic D , Ryan J *et al*. Socioeconomic, Behavioural, and social health correlates of optimism and pessimism in older men and women: a cross‐sectional study. Int J Environ Res Public Health 2023; 20: 3259. 36833951 10.3390/ijerph20043259 PMC9961087

[13] Bondevik M , Skogstad A . Loneliness, religiousness, and purpose in life in the oldest old. J Religious Gerontol 2000; 11: 5–21.

[14] Hajek A , Lehnert T , Wegener A , Riedel‐Heller SG , König H‐H . Factors associated with preferences for long‐term care settings in old age: evidence from a population‐based survey in Germany. BMC Health Serv Res 2017; 17: 1–9. 28222774 10.1186/s12913-017-2101-y PMC5320639

[15] Hajek A , Lehnert T , Wegener A , Riedel‐Heller SG , Koenig H‐H . Long‐term care preferences among individuals of advanced age in Germany: results of a population‐based study. Gesundheitswesen (Bundesverband Der Arzte Des Offentlichen Gesundheitsdienstes (Germany)) 2017; 80: 685–692. 28268234 10.1055/s-0042-124663

[16] Koren C . Men's vulnerability–women's resilience: from widowhood to late‐life repartnering. Int Psychogeriatr 2016; 28: 719–731. 26691683 10.1017/S1041610215002240

[17] Cullen MR , Baiocchi M , Eggleston K , Loftus P , Fuchs V . The weaker sex? Vulnerable men and women's resilience to socio‐economic disadvantage. SSM‐Popul Health 2016; 2: 512–524. 29349167 10.1016/j.ssmph.2016.06.006 PMC5757782

[18] Gierveld JDJ , Tilburg TV . A 6‐item scale for overall, emotional, and social loneliness: confirmatory tests on survey data. Res Aging 2006; 28: 582–598.

[19] Bude H , Lantermann E‐D . Soziale exklusion und exklusionsempfinden. KZfSS Kölner Z Soziol Sozialpsychol 2006; 58: 233–252.

[20] Peltzer K , Pengpid S . Loneliness correlates and associations with health variables in the general population in Indonesia. Int J Ment Health Syst 2019; 13: 1–11. 31007711 10.1186/s13033-019-0281-z PMC6456978

[21] Pengpid S , Peltzer K . Prevalence and associated factors of incident and persistent loneliness among middle‐aged and older adults in Thailand. BMC Psychol 2023; 11: 70. 36918991 10.1186/s40359-023-01115-4 PMC10015912

[22] Pengpid S , Peltzer K , Anantanasuwong D . Longitudinal associations of loneliness with mental ill‐health, physical ill‐health, lifestyle factors and mortality in ageing adults in Thailand. BMC Psychiatry 2023; 23: 855. 37978470 10.1186/s12888-023-05263-0 PMC10656829

[23] Hajek A , König H‐H . Which factors contribute to loneliness among older Europeans? Findings from the survey of health, ageing and retirement in Europe: determinants of loneliness. Arch Gerontol Geriatr 2020; 89: 104080. 32371343 10.1016/j.archger.2020.104080

[24] Hajek A , Volkmar A , König H‐H . Prevalence and correlates of loneliness and social isolation in the oldest old: a systematic review, meta‐analysis and meta‐regression. Soc Psychiatry Psychiatr Epidemiol 2023; 60: 1–23. 10.1007/s00127-023-02602-0 PMC12119783 38102477

[25] UNESCO . International Standard Classification of Education. ISCED 1997, Re‐edition edn. Paris: UNESCO, 2006.

[26] Ware JJ , Sherbourne C . The MOS 36‐item short‐form health survey (SF‐36). I. Conceptual framework and item selection. Med Care 1992; 30: 473–483. 1593914

[27] Radloff LS . The CES‐D scale: a self‐report depression scale for research in the general population. Appl Psychol Measur 1977; 1: 385–401.

[28] Enders CK . The performance of the full information maximum likelihood estimator in multiple regression models with missing data. Educ Psychol Meas 2001; 61: 713–740.

[29] Shaw BP . Meeting assumptions in the estimation of reliability. Stata J 2021; 21: 1021–1027.

[30] Wagner AJ , Reifegerste D . Real men don't talk? Relationships among depressiveness, loneliness, conformity to masculine norms, and male non‐disclosure of mental distress. SSM‐Mental Health 2024; 5: 100296.

[31] Hajek A , Sutin A , Luchetti M *et al*. Perception of one's social environment and loneliness: results of the nationally representative “old age in Germany (D80+)” study. Soc Psychiatry Psychiatr Epidemiol 2024; 60: 1–8. 39354148 10.1007/s00127-024-02774-3 PMC12119379

[32] Teneva N , Lemay EP Jr . Projecting loneliness into the past and future: implications for self‐esteem and affect. Motivation Emotion 2020; 44: 772–784.

[33] Hajek A , König H‐H . How do individuals rate their health compared to others? Findings based on a nationally representative sample in Germany. BMC Public Health 2024; 24: 197. 38229062 10.1186/s12889-023-17600-9 PMC10792948

